# Real-time PCR assay for the diagnosis of pleural tuberculosis

**Published:** 2017-06-30

**Authors:** Martha Alejandra Casallas-Rivera, Ana María Cárdenas Bernal, Luis Fernando Giraldo-Cadavid, Enrique Prieto Diago, Sandra Paola Santander

**Affiliations:** 1 Universidad de la Sabana. Chía, Colombia; 2 Internal Medicine, Hospital Universitario de la Samaritana. Bogotá, Colombia

**Keywords:** Diagnostic tests, pleural effusion, Mycobacterium tuberculosis, tuberculosis pleural, validation studies, Real-Time Polymerase Chain Reaction, Ziehl-Neelsen, likelihood ratios

## Abstract

**Introduction:**

The diagnosis of pleural tuberculosis requires an invasive and time-consuming reference method. Polymerase chain reaction (PCR) is rapid, but validation in pleural tuberculosis is still weak.

**Objective:**

To establish the operating characteristics of real-time polymerase chain reaction (RT-PCR) hybridization probes for the diagnosis of pleural tuberculosis.

**Methods:**

The validity of the RT-PCR hybridization probes was evaluated compared to a composite reference method by a cross-sectional study at the Hospital Universitario de la Samaritana. 40 adults with lymphocytic pleural effusion were included. Pleural tuberculosis was confirmed (in 9 patients) if the patient had at least one of three tests using the positive reference method: Ziehl-Neelsen or *Mycobacterium* tuberculosis culture in fluid or pleural tissue, or pleural biopsy with granulomas. Pleural tuberculosis was ruled out (in 31 patients) if all three tests were negative. The operating characteristics of the RT-PCR, using the Mid-P Exact Test, were determined using the OpenEpi 2.3 Software (2009).

**Results:**

The RT-PCR hybridization probes showed a sensitivity of 66.7% (95% CI: 33.2%-90.7%) and a specificity of 93.5% (95% CI: 80.3%-98.9%). The PPV was 75.0% (95% CI: 38.8%-95.6%) and a NPV of 90.6% (95% CI: 76.6%-97.6%). Two false positives were found for the test, one with pleural mesothelioma and the other with chronic pleuritis with mesothelial hyperplasia.

**Conclusions:**

The RT-PCR hybridization probes had good specificity and acceptable sensitivity, but a negative value cannot rule out pleural tuberculosis.

## Resumen

**Introducción:**

El diagnóstico de tuberculosis pleural requiere un método de referencia invasivo y demorado. La reacción en cadena de la polimerasa es rápida, pero su validación en tuberculosis pleural aún es débil.

**Objetivo:**

Establecer las características operativas de la reacción en cadena de la polimerasa en tiempo real (RT-PCR) sondas de hibridación para el diagnóstico de tuberculosis pleural.

**Métodos:**

Se evaluó la validez de la RT-PCR sondas de hibridación comparada con un método de referencia compuesto mediante un estudio transversal en el Hospital Universitario de la Samaritana. Se incluyeron 40 adultos con derrame pleural linfocitario. Tuberculosis pleural fue confirmada (en 9 pacientes) si el paciente tenía mínimo una de tres pruebas del método de referencia positiva: Ziehl-Neelsen o cultivo para *Mycobacterium tuberculosis* en líquido o tejido pleural, o biopsia pleural con granulomas; se descartó tuberculosis pleural (en 31 pacientes) si las tres pruebas eran negativas. Se determinaron las características operativas de la RT-PCR, mediante la Prueba Mid-P Exact, con el Software OpenEpi 2.3 (2009).

**Resultados:**

La RT-PCR sondas de hibridación mostró una sensibilidad del 66.7% (IC 95%: 33.2%-90.7%) y una especificidad del 93.5% (IC 95%: 80.3%-98.9%). El VPP fue de 75.0% (IC 95%: 38.8%-95.6%) y un VPN de 90.6% (IC 95%: 76.6%-97.6%). Se encontraron dos falsos positivos para la prueba, uno con mesotelioma pleural y otro con pleuritis crónica con hiperplasia mesotelial.

**Conclusiones:**

La RT-PCR sondas de hibridación tuvo una buena especificidad y una aceptable sensibilidad, pero un valor negativo no puede descartar tuberculosis pleural.

## Introduction

Tuberculosis (TB) has been a major public health disease for decades ^1^. The effects may be multisystemic and when it compromises spaces with paucibacillary behavior, it becomes more difficult to diagnose and to initiate appropriate and timely treatment ^2^. 

Epidemiologically, TB has a worldwide distribution and the mortality rate of 2014 was reduced to about half that of 1990 ^3^. However, in 2015, 1.4 million people died of TB worldwide ^4^. Global incidence of TB has declined by 1.5% per year since 2000, and 18% total ^3^. In 2015 Colombia reported an incidence of 31 cases per 100,000 inhabitants, with a mortality of 2.1 cases per 100,000 inhabitants ^4^. Of these, 2,385 cases were extra pulmonary, of which 863 corresponded to pleural tuberculosis (TBP) ^5^. TB is, together with HIV, one of the leading causes of death in the world ^4^. 

The detection of bacillus by culture, staining of histopathological studies or body fluids is essential for the diagnosis of TB, but it is necessary to take into account that less than 5% of the cases of TBP show a positive smear microscopy in the liquid Pleural due to the paucibacillary naturalization of this entity ^6^. Identification of the species and its resistance to antibiotics is achieved by culture and/or PCR-based techniques ^7^. *Mycobacterium tuberculosis* is isolated in culture in only 20% to 40% of cases of confirmed tuberculous pleuritis, the diagnostic yield of the culture being low in pleural fluid, but may be increased if a sample of pleural tissue is also cultured ^6^ ^,^ ^8^ ^,^ ^9^. Pleural biopsy with evidence of granulomas is positive in 75% of cases ^6^ ^,^ ^8^. 

Adenosine deaminase (ADA) nonspecific inflammatory marker is an enzyme the activity of which is involved in the differentiation and proliferation of lymphocytes and the activation of macrophages and neutrophils, being indicative of active local inflammatory response ^6^. The determination of the ADA2 isoenzyme could increase the precision since it is released from monocytes and is found in a high concentration in pleuritis by TB. It has been suggested that higher levels of ADA in pleural fluid better predict the diagnosis of TB with a sensitivity of 90 to 100% and a specificity of 89 to 100% when using the Guisti method ^6^. The specificity to discriminate between pleural effusion due to TB and malignancy was 95%, being low for the differentiation between paraneumonic effusion ^6^.

Consequently, a combination of several tests, including cultures and Ziehl-Neelsen (ZN) staining of the fluid or pleural tissue, has been recommended as a reference method for the diagnosis of TBP, and the histopathological study of the pleural tissue ^6^ ^,^ ^8^. 

The slow growth of *M. tuberculosis* in culture has led to the search for rapid diagnostic tests and new methods that detect it directly in clinical samples, without the need to wait for the result of such cultures ^10^. 

Therefore, other diagnostic methods have been used, including the real-time polymerase chain reaction (RT-PCR) of the qualitative type, which is a molecular biology test, which identifies the DNA of the mycobacteria, using a technique where the amplification and detection processes occur simultaneously in the same closed vial, with no need for any subsequent action, emitting results in less time and with a high specificity (98%), but with a low or variable sensitivity (62%) not being useful to exclude the disease ^11^. 

The fluorescence detection systems used in RT-PCR can be of two types: intercalators and specific probes labeled with fluorochromes ^11^. Intercalating agents are fluorochromes that significantly increase the emission of fluorescence when bound to double-helix DNA. The most used in RT-PCR is SYBR Green I ^11^. The main drawback is low specificity, because they bind indistinctly to nonspecifically generated products or primer dimers, which are very common in the polymerase chain reaction (PCR). To improve specificity, optimum reaction conditions and careful selection of primers should be employed to decrease the risk of dimer formation. In addition, it is advisable to initiate the DNA synthesis reaction at high temperatures (*hot-start PCR*), which significantly reduces the risk of nonspecific amplifications ^11^.

Specific hybridization probes are probes labeled with two types of fluorochromes, a donor and an acceptor. The process is based on the transfer of fluorescent energy by resonance (FRET) between the two molecules. The most used are ^11^:

1. Hydrolysis probes, also referred to as TaqMan probes

2. Molecular beacons.

3. FRET probes. The system consists of two probes that bind to adjacent sequences of the target DNA. One of the probes carries a donor at the 3' end and the other an acceptor at the 5' end. When the probes are hybridized, the two fluorochromes are close. Upon being energized, the donor transfers its energy to the acceptor which, in turn, emits the fluorescence that the reader of the equipment detects.

In all these systems, the increase of DNA in each cycle corresponds to an increased hybridization of the probes, which leads to an increase in the same proportion of emitted fluorescence. The use of probes guarantees specificity of the detection and it allows the identification of polymorphisms or point mutations, but the cost is higher than SYBR Green and optimization of the reaction conditions is more difficult ^11^.

The diagnostic test evaluated in this work is *M. tuberculosis* Real-TM (RT-PCR kit for *M. tuberculosis* complex detection) which is a real-time amplification test for the qualitative detection of *M. tuberculosis* complex in biological materials (the result is obtained in an average of 3.5 days). DNA is extracted from the samples, amplified and detected by fluorescent probes specific for *M. tuberculosis* and *M. tuberculosis* (internal control). *M. tuberculosis* (internal control) is an IS6110 insertion DNA fragment of *MTb* modified and cloned into the bacteriophage (, containing DNA fragments used in the kit as matrix for the primer.

In this regard, different PCR studies have been published for the diagnosis of TBP, where handmade and commercial PCR tests have been used, with the use of different kits that do not make the results very homogeneous ^12^. In assessing these studies, oscillating values of mean specificity greater than 90% were found for PCR, but with mean sensitivity less than 80% ^12^ according to the type of test used ^12^ ^-^ ^15^.

Up to 2012 two RT-PCR studies were found, both published in 2011, one of which was published by Kalantri *et al*. ^16^, comparing RT-PCR with INFγ, IgA and ADA with respect to a combined reference method, where they found a sensitivity of 80%, higher than that mentioned in the other studies and a specificity of 98%. However, when compared to other criteria (clinical manifestations, response to empirical treatment and exclusion of other diagnoses) instead of the composite reference method, this sensitivity decreased to 64.9% while maintaining specificity unchanged ^15^. The other study, developed in Brazil, by Rosso*,* et al*.* ^17^, reported a sensitivity of 42.8% and a specificity of 94.2% for this test ^17^. 

Considering that the evidence on the validity of RT-PCR in TBP is insufficient, we decided to carry out a study to evaluate the validity of this test comparing it with a combined reference method, in the adult hospitalization service of the Hospital Universitario de la Samaritana (HUS), in patients with lymphocytic exudate pleural effusion (according to light criteria and with a higher proportion of lymphocytes than neutrophils). 

## Materials and Methods

### Design

Study of cross-sectional diagnostic tests to determine the validity of pleural fluid RT-PCR for the diagnosis of TBP.

### Participants

The population under study were all patients over 18 years of age evaluated for lymphocytic exudate pleural effusion during the period from September 1, 2009 to September 30, 2012, in the HUS internal medicine hospitalization service, where the patient and the attending physician agree to participate in the study of pleuritis with the standard methods used by them.

Inclusion criteria were: Patients older than 18 years of age hospitalized in the HUS internal medicine service during the study period, with lymphocytic exudate-type pleural effusion (ratio of fluid proteins to serum proteins greater than 0.5 or LDH ratio of the fluid over serum LDH greater than 0.6 or LDH of the fluid greater than 2/3 of the upper limit of the serum normal value and with more than 50% of lymphocytes ^18^ with no identified aetiology the study of which included ZN of pleural fluid or tissue, culture of pleural fluid or pleural tissue for *M. tuberculosis* and/or histology of pleural tissue with granulomas and where RT-PCR was performed with liquid or pleural tissue hybridization probes for *M. tuberculosis.*

Exclusion criteria were: Transudated pleural effusion (does not meet any of the exudate criteria discussed above) ^18^; Neutrophilic exudate pleural effusion (meets exudate criteria above and with more than 50% neutrophils); Patients whose samples have been processed using techniques other than the protocols described later in this document. Patients for whom the study of the etiology of pleural effusion was incomplete because they had negative results in an insufficient number of standard diagnostic tests for an exudate pleural lymphocyte (they were not studied with all the tests that are part of the reference method of this work despite having negative results in the tests that were performed: ZN of pleural fluid or tissue, fluid culture or pleural tissue for *M. tuberculosis* and histology of pleural tissue).

Initially we used the molecular biology laboratory database where we had all the RT-PCR hybridation probes for *M. tuberculosis* performed in pleural liquid or tissue during the years of the study and with this information we arranged to search (previous authorization from the Patient and hospital) in the medical records of these patients the other inclusion criteria required.

Once the information was collected, we prepared to perform their respective coding and tabulation by double entry, using an instrument designed for data collection.

### Diagnostic methods and reference method

A combined reference method was defined as follows: 1. Culture of pleural fluid or tissue for *M. tuberculosis*; 2. Ziehl-Neelsen (ZN) of pleural fluid or tissue; and 3. Histology of pleural tissue. 

One of these three positive tests diagnosed *M. tuberculosis* pleuritis and it was ruled out if all three were negative. It was considered a probable case if the reference method was negative but the RT-PCR hybridization probes for *M. tuberculosis* was positive and the ADA greater than 47 UI/L ^6^. 

Both the reference method tests and the ADA and the RT-PCR hybridization probes for *M. tuberculosis* were performed simultaneously once the diagnosis was made from lymphocyte exudate initially in pleural fluid and if these were inconclusive for a definitive diagnosis, these tests were made simultaneously in a sample of pleural biopsy. At the beginning of the study, there was no diagnosis of the etiology of the pleural effusion or previous history of TB or cancer, although this was not an exclusion criterion. 

The complete extraction of the DNA in the pleural fluid of the patients, as well as the assembly of the RT-PCR hybridization probes was performed according to the recommendations of the kit manufacturer (SACACE Biotechnologies, Italy). In order to remove inhibitors from the RT-PCR reaction, initially all the pleural fluids obtained were treated with 250 mg of N-acetyl-L-Cysteine (Sigma), 4% NaOH and sodium citrate at 2.94%. The DNA subsequently obtained was used to assemble the RT-PCR using hybridization probes (FAM Emission) for the qualitative detection of the IS6110 gene specifically present in the Mycobacteria DNA of the tuberculous complex (*M. tuberculosis, M. Africanum, M. bovis, M. bovis BCG, M. microti*). Under the following amplification conditions: 1 Cycle: 95° C for 15 min, 40 Cycles: 95° C for 15 s, 65° C for 30 s, 72° C for 15 s. In order to discard the false negatives by the presence of amplification reaction inhibitors, an internal control (IC) of amplification (JOE emission) was added to all the samples. The assembly of the RT-PCR and the analysis of the results was carried out using the STRATAGENE MX 3005P equipment in the MxPro program. Samples were considered positive or negative based on increased fluorescence above the threshold established in the FAM and JOE channels. Thus, a sample was considered positive for *M. tuberculosis* if the fluorescence exceeded the limit of the established threshold in the FAM (green) channel, whereas a sample was considered negative if it did not show fluorescence in the FAM channel and presented positive fluorescence in the JOE channel. It is important to clarify that the results obtained for each patient with this technique were obtained in a maximum time of four hours.

All samples included in the study were grown in Ogawa kudot solid medium, using the modified Petroff technique, where the liquid samples were centrifuged at 3,000 rpm for 30 min and the sediment was resuspended in a maximum of 2 mL and deposited in the medium directly, cultivating in an inclined position at 37° C for 8 weeks.

These tests were processed by HUS personnel with extensive track record and experience in molecular biology, microbiology, clinical laboratory and pathology, respectively. The persons responsible for carrying out the tests of the reference method did not know the result of RT-PCR for *M. Tuberculosis*. And the person in charge of performing RT-PCR for *M. tuberculosis* was unaware of the outcome of the reference method.

### Statistical Methods 

The sample size estimation was based on the likelihood ratios and confidence intervals in a single-sample diagnostic test, where diagnostic tests are compared in the same subjects (paired design), for a 95% expected sensitivity, a 95% expected specificity, a 50% prevalence of pleural tuberculosis in the studied population, expecting an amplitude of the confidence interval of 95% of 0.1 on each side, using the equation proposed by Duffau ^19^ - ^21^. With these parameters a sample size of 34 patients was estimated for this study.

Sensitivity, specificity, positive and negative predictive values, and respective confidence intervals were calculated for RT-PCR, ZN coloration and culture in pleural fluid, compared to the reference method. Confidence intervals were determined using the Mid-P Exact Test. The software OpenEpi version 2.3 (2009) was used.

## Results

### Participants and results

A population of 60 patients with pleural effusion in the HUS was included during the study period, of whom 40 met the inclusion criteria and of these nine (prevalence of 22.5%) were confirmed the diagnosis of TBP with the method reference. Of these nine patients confirmed with TBP, six were positive and three negative for RT-PCR, the latter were considered false negatives of the RT-PCR, since they presented resolution of the picture with antituberculous treatment. There were no patients with probable TBP (probable TBP: negative reference method with positive RT-PCR and ADA greater than 47 IU/L). There were two false positives for RT-PCR, one with pleural mesothelioma and the other with chronic pleuritis with mesothelial hyperplasia. In patients diagnosed with TPB, we found an average of 47.4 years of age with a standard deviation of ( 15.2. 66.7% of the patients with TBP were men.

**Figure 1:**
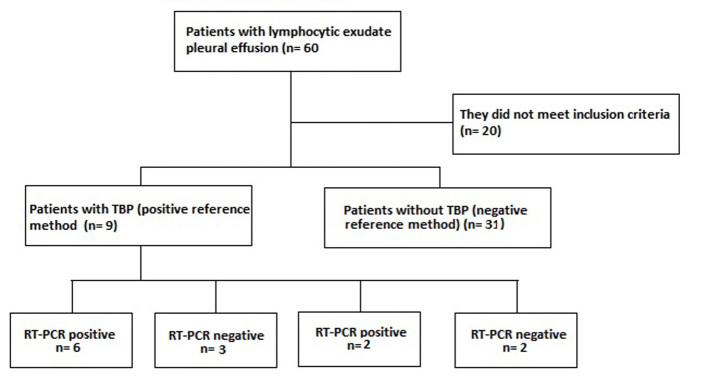
Flowchart: RT - PCR in pleural fluid for diagnosis of TBP. Note: TBP: pleural tuberculosis; RT-PCR: real-time polymerase chain reaction

Of the 40 patients included in the study (Fig. 1), 9 patients were confirmed TBP: 1 per culture for TB, ZN and PCR for TB positive in pleural fluid; 1 by ZN and PCR for TB positive in pleural fluid; 2 by culture for TB and PCR for positive TB in pleural fluid; 1 per culture for TB and PCR for positive TB in pleural fluid and pleural biopsy with casein granulomas; 1 per culture for positive TB in pleural fluid, culture for TB and PCR for positive TB in pleural tissue and pleural biopsy with casean granulomas; 2 by pleural biopsy with casein granulomas; 1 by PCR for positive TB in pleural fluid and pleural biopsy with casein granulomas. TB was discarded in thirty-one patients because of the negative reference method, where the cause of pleural lymphocytic effusion in 29.0% was due to association with neoplasia by pleural biopsy, of which three cases were due to mesothelioma and the remaining cases due to metastases, squamous cell carcinoma and adenoma without specified primary. 58.1% were associated with chronic reactive pleural inflammation with no other etiology and 12.9% had no clear diagnosis at the time of inclusion in the study.

### Estimates

A sensitivity of 66.7% (95% CI: 33.2%-90.7%) and a specificity of 93.5% (95% CI: 80.3%-98.9%) were calculated for the test under study (qualitative RT-PCR for *MTb*). The PPV was 75% (95% CI: 38.8%-95.6%) and the NPV was 90.6% (95% CI: 76.6%-97.6%) (Table 1).

**Table 1:** Results in the different diagnostic tests with respect to the diagnosis of TBP according to the definitive diagnosis obtained by the reference method.

Definitive Diagnosis	N: 40	RT - PCR in liquid		Liquid ZN		Tissue ZN		MTb liquid culture		MTb Tissue Culture		Histology of TBP pleura	
		Positive	Negative	Positive	Negative	Positive	Negative	Positive	Negative	Positive	Negative	Positive	Negative
TBP	9	6	3	2	3	-	2	5	2	1	-	5	-
Mesothelioma	5	1	4	-	5	-	-	-	5	-	-	-	5
Chronic pleuritis with mesothelial hyperplasia	2	1	1	-	2	-	1	-	2	-	-	-	2
Other Tumors	5	-	5	-	5	-	-	-	5	-	-	-	5
Nonspecific Pleuritis	19	-	19	-	17	-	2	-	18	-	1	-	19
Total	40	8	32	2	32	-	5	5	32	1	1	3	31

TBP: pleural tuberculosis; RT-PCR: real-time polymerase chain reaction; ZN: coloration of Ziehl Neelsen; MTb: Mycobacterium tuberculosis.

With regard to culture for *M. tuberculosis* in pleural fluid, a sensitivity of 55.6% (95% CI: 39.7%-89.2%) was found with a specificity of 100% (95% CI: 87.1-100%); and for ZN in pleural fluid a sensitivity of 22.2% (95% CI: 39.7%-89.2%) was calculated with a specificity of 100% (95% CI: 87.1%-100%) (Table 1).

## Discussion

With these results, the operating characteristics of the RT-PCR for the diagnosis of *M. tuberculosis* in pleural fluid in the HUS were established. A sensitivity (66.7%) was found, similar to that found in previously referenced studies ^12^ - ^14^ ^.^ ^16^. On the other hand, the specificity calculated was relatively high (93.5%) and both results were similar to that found by other authors. 

In culture for *M. tuberculosis* in pleural fluid a sensitivity of 55.6% was found while ZN in pleural fluid was 22.2%; both had a specificity of 100%. These values show lower sensitivities than the RT-PCR, confirming once again the need to have a combined reference method since these tests alone could not rule out the presence of pleural tuberculosis. Additionally, in the case of cultures, the time required to obtain the diagnosis is extended to 8 weeks in such a way that a rapid test is required, such as RT-PCR, to define the initial management of the patient in a maximum time of four hours which is what this test would take to generate a result.

In the meta-analysis performed by Pai*,* et al*.* ^12^ *,* commercial and artisanal PCR were evaluated, reporting a sensitivity of 62% and 71%, with a specificity of 98% and 93%, respectively, which is similar to that found in our research for RT-PCR. The studies included in this meta-analysis had small sample sizes and were heterogeneous; the reference method used included culture for *M. tuberculosis*, clinical findings, microbiology or biopsy, so that these reference methods lack precision to make a definitive diagnosis and make validation of the test under study difficult. Later in 2005, Chakravorty *et al*. ^13^, used the IS6110 and devRf3 (artesian tests) in liquid with a sensitivity of 75.5% and a specificity of 93.8%, similar for each test, with a greater sensitivity to that reported by us and with the same specificity, with a method of compound reference, not well defined, given by clinical, microbiology, cytology/histology and response to treatment, with a sample of 87 patients. In Taiwan, Liu *et al.* ^14^, published an artisanal PCR study using the IS6110 segment, reporting a sensitivity of 43.3% and a specificity of 95.5%, being the sensitivity lower than that described in our research, with no variation in the specificity and with false positives in 4.5% of the patients studied. Additionally, in the combined reference method, clinical and response to empirical treatment were included, making it difficult to perform an objective evaluation. And finally Kalantri, *et al*. ^16^, with a population of 204 cases divided into three groups by their diagnostic form (confirmed, probable and without TBP) reported a sensitivity of 80% having a composite reference method equal to that used by us and of 57.7% taking into account the clinic, response to treatment and having excluded other pathologies, with a specificity of 98.0% similar to that described in this study. 

According to our reference method, there were 9 cases of TBP in our study and 31 cases with lymphocytic pleuritis due to causes other than TB, which gives us a prevalence of TBP of 23%. This prevalence is similar to that found in TBP studies in Spain ^17^ and higher than the prevalence of TB in the studies carried out in the majority of countries with GDP higher than in our country ^12^ and this could be explained by an epidemiological transition in our population with an increase in the frequency of neoplastic diseases and a reduction in the frequency of infectious diseases.

The operating characteristics of the PCR vary depending on the technique used and the association of other diagnostic tests and clinical characteristics of the patient. It can be observed that the use of RT-PCR requires less time, with high specificity but with low sensitivity. This makes the test insufficient to rule out TBP if the RT-PCR is negative and there is no alternative diagnosis and it requires performing the complete combined reference method. By having sensitivity and specificity values superior to those obtained with the other tests individually and yielding results much more quickly, it is a useful test to define early on the treatment to follow with a patient with lymphocyte exudate: if it is positive there would be valid reasons to start anti-tuberculosis treatment and if it is negative, not do so. However, these results should be analyzed in the clinical context of the patient and should be confirmed with reference method (ZN, culture and biopsy), since the predictive values are not good enough (PPV: 75.0% and NPV: 90.6%) for the use of RT-PCR as a definitive diagnostic method. In fact, two false positives were presented with a diagnosis of mesothelioma and chronic pleuritis with mesothelial hyperplasia, respectively.

For this study, a combined reference method was used, which allowed for a definitive and objective diagnosis of TBP and, therefore, to assess a more precisely the operating characteristics of the test under study, becoming a strength for this research. However, we should note that a weakness of the study is the sample size, which was low for the findings finally found, which increased confidence intervals and decreased study accuracy. This forces us to suggest conducting studies of validation of the RT-PCR with larger sample sizes, to increase the precision of the results.

## Conclusions

The RT-PCR for *M. tuberculosis* in pleural fluid has good specificity for the diagnosis of TBP, but because its predictive values are not high enough, it should not be used for the definitive diagnosis of the disease. Its greatest use would be as a method to quickly define the behavior to follow with a patient, while the reports of the definitive diagnostic tests arrive. Cultures for *M. tuberculosis* and ZN of pleural fluid and tissue have a lower diagnostic yield than RT-PCR and it is necessary to continue using the combined reference method to establish the definitive diagnosis of lymphocytic pleuritis. 

It is necessary to carry out new studies with a larger sample size in order to increase the precision of the previously established results.
