# Supplementary material for: Real-time PCR assay for the diagnosis of pleural tuberculosis
Source: Colomb Med (Cali). 2017 Jun 30;48(2):47–52. (PMC5625560)
Supplement: Translation

# Translation: Reacción en cadena de la polimerasa en tiempo real para el diagnóstico de tuberculosis pleural

## Introducción

La tuberculosis (TB) es una enfermedad de gran impacto en salud pública desde hace décadas ^1^. Su compromiso puede ser multisistémico y cuando compromete espacios en donde su comportamiento es paucibacilar, se hace más difícil su diagnóstico y el inicio de un tratamiento adecuado y oportuno ^2^. 

Epidemiológicamente, la TB tiene distribución mundial, la tasa de mortalidad de 2014 se redujo a cerca de la mitad de la de 1990 ^3^. No obstante, en el 2015 murieron de TB 1.4 millones de personas en todo el mundo ^4^. La incidencia mundial de la TB ha disminuido un 1.5% al año desde el 2000, y un 18% en total ^3^. Colombia en el año 2015 reportó una incidencia de 31 casos por 100,000 habitantes, con una mortalidad de 2.1 casos por 100,000 habitantes ^4^. De éstos, 2,385 casos fueron extra pulmonares de los cuales 863 correspondieron a tuberculosis pleural (TBP) ^5^. La TB constituye, junto con el VIH, una de las principales causas mundiales de muerte ^4^. 

Para el diagnóstico de la TB es fundamental la detección del bacilo por cultivo, coloraciones de estudios histopatológicos o de líquidos corporales, pero hay que tener en cuenta que en menos del 5% de los casos de TBP se logra obtener una baciloscopia positiva en el líquido pleural debido a la naturaliza paucibacilar de esta entidad ^6^. La identificación de la especie y su resistencia a antibióticos se consigue mediante cultivo y/o técnicas basadas en PCR ^7^. El *Mycobacterium tuberculosis* se aísla en cultivo sólo en un 20 a 40% de los casos de una pleuritis tuberculosa confirmada, siendo el rendimiento diagnóstico del cultivo en líquido pleural bajo, pero puede aumentar si también se cultiva una muestra de tejido pleural ^6^^,^^8^^,^^9^. La biopsia pleural con evidencia de granulomas es positiva en un 75% de los casos ^6^^,^^8^. 

La Adenosin deaminasa (ADA) marcador inflamatorio no específico, es una enzima cuya actividad está involucrada en la diferenciación y proliferación de los linfocitos y la activación de los macrófagos y neutrófilos, siendo indicativa de respuesta inflamatoria local activa ^6^. La determinación de la isoenzima ADA2 podría aumentar la precisión ya que es liberada de los monocitos y es encontrada en una alta concentración en pleuritis por TB. Se ha sugerido que niveles mayores de ADA en líquido pleural predicen mejor el diagnóstico de TB con una sensibilidad del 90 al 100% y una especificidad del 89 al 100% cuando se usa el método Guisti ^6^. La especificidad para discriminar entre derrame pleural por TB y malignidad fue del 95%, siendo baja para la diferenciación entre derrame paraneumónico ^6^.

Por estos motivos se ha recomendado utilizar como método de referencia para hacer el diagnóstico de TBP la combinación de varias pruebas, incluyendo los cultivos y la tinción de Ziehl-Neelsen (ZN) del líquido o del tejido pleural; y el estudio histopatológico del tejido pleural ^6^^,^^8^.

El lento crecimiento del *M. tuberculosis* en cultivo ha llevado a la búsqueda de pruebas rápidas de diagnóstico y de nuevos métodos que lo detecten directamente en muestras clínicas, sin la necesidad de esperar el resultado de dichos cultivos ^10^. 

Por esta razón se han venido utilizando otros métodos diagnósticos, entre los cuales está la reacción en cadena de la polimerasa en tiempo real (RT-PCR) de tipo cualitativa, que es una prueba de biología molecular, que identifica el ADN de la micobacteria, utilizando una técnica donde los procesos de amplificación y detección se producen de manera simultánea en el mismo vial cerrado, sin necesidad de ninguna acción posterior, emitiendo resultados en menor tiempo y con una alta especificidad (98%), pero con una baja o variable sensibilidad (62%) no siendo útil para excluir la enfermedad ^11^.

Los sistemas de detección por fluorescencia empleados en la RT-PCR pueden ser de dos tipos: agentes intercalantes y sondas específicas marcadas con fluorocromos ^11^. Los agentes intercalantes son fluorocromos que aumentan notablemente la emisión de fluorescencia cuando se unen a ADN de doble hélice. El más empleado en RT - PCR es el SYBR Green I ^11^. El principal inconveniente es su baja especificidad, debido a que se unen de manera indistinta a productos generados inespecíficamente o a dímeros de cebadores, muy frecuentes en la reacción en cadena de la polimerasa (PCR). Para mejorar la especificidad se deben emplear condiciones de reacción óptimas y una selección cuidadosa de los cebadores para disminuir el riesgo de formación de dímeros. Además, es recomendable iniciar la reacción de síntesis de ADN a temperaturas elevadas *(hot-start PCR)* lo cual disminuye de forma notable el riesgo de amplificaciones inespecíficas ^11^.

Las sondas de hibridación específicas, son sondas marcadas con dos tipos de fluorocromos, un donador y un aceptor. El proceso se basa en la transferencia de energía fluorescente mediante resonancia (FRET) entre las dos moléculas. Las más utilizadas son ^11^:

1. Sondas de hidrólisis, denominadas también sondas TaqMan

2. Molecular beacons*.*

3. Sondas FRET. El sistema se compone de dos sondas que se unen a secuencias adyacentes del ADN diana. Una de las sondas lleva un donador en el extremo 3' y la otra un aceptor en el extremo 5'. Cuando las sondas están hibridadas, los dos fluorocromos están próximos. Al ser excitado, el donador transfiere su energía al aceptor que, a su vez, emite la fluorescencia que detecta el lector del equipo.

En todos estos sistemas, el incremento de ADN en cada ciclo se corresponde con un aumento de hibridación de las sondas, lo que conlleva un aumento en la misma proporción de fluorescencia emitida. El empleo de sondas garantiza la especificidad de la detección y permite identificar polimorfismos o mutaciones puntuales, pero su coste es más elevado que el SYBR Green y la optimización de las condiciones de la reacción resulta más difícil ^11^.

La prueba diagnóstica evaluada en el presente trabajo es *M. tuberculosis* Real-TM (Equipo de RT-PCR para la detección del complejo *M. tuberculosis*) la cual es un test de amplificación en tiempo real para la detección cualitativa del complejo *M. tuberculosis* en materiales biológicos (se obtiene el resultado en un promedio de 3.5 días). El ADN es extraído de las muestras; amplificado y detectado mediante sondas fluorescentes específicas para *M. tuberculosis* y control interno (*M. tuberculosis*). *Mycobacterium tuberculosis* (control interno) es un fragmento de ADN de inserción IS 6110 de *MTb* modificado y clonado en el bacteriófago (, conteniendo fragmentos de ADN usados en el equipo como matriz para el primer.

Al respecto, se han publicado diferentes estudios de PCR para el diagnóstico de TBP, en los cuales se han utilizado pruebas de PCR artesanales y otras comerciales, con el uso de diferentes estuches que han hecho que los resultados no sean muy homogéneos ^12^. Al valorar dichos estudios se ha encontrado para la PCR valores oscilantes de especificidad promedio mayor al 90%, pero con sensibilidad promedio menor al 80% ^12^ según el tipo de prueba utilizada ^12^^-^^15^.

Hasta el año 2012 se encontraron dos estudios con RT-PCR, ambos publicados en el año 2011, uno de ellos, publicado por Kalantri, *et al*. ^16^, quienes comparan la RT- PCR con el INFγ, IgA y ADA con respecto a un método de referencia combinado, donde encontraron una sensibilidad del 80%, superior a la mencionada en los demás estudios y una especificidad del 98%. Sin embargo, cuando se comparaba respecto a otros criterios (manifestaciones clínicas, respuesta a tratamiento empírico y la exclusión de otros diagnósticos) en lugar del método de referencia compuesto, esta sensibilidad disminuía al 64.9% manteniendo una especificidad sin cambios ^15^. El otro estudio, desarrollado en Brasil, por Rosso*, et al.* ^17^, reportó una sensibilidad del 42.8% y una especificidad del 94.2% para esta prueba ^17^. 

Considerando que la evidencia sobre la validez de la RT - PCR en TBP es insuficiente, se decidió realizar un estudio para evaluar la validez de esta prueba comparándola con un método de referencia combinado, en el servicio de hospitalización adultos, del Hospital Universitario de la Samaritna (HUS), en pacientes con derrame pleural tipo exudado linfocitario (según los criterios de light y con mayor proporción de linfocitos que de neutrófilos). 

## Materiales y Métodos

### Diseño

Estudio de pruebas diagnósticas de corte transversal, para determinar la validez de la RT-PCR en líquido pleural para el diagnóstico de TBP.

### Participantes

La población en estudio fueron todos los pacientes mayores de 18 años evaluados por derrame pleural tipo exudado linfocitario, durante el periodo comprendido entre el primero de septiembre de 2009 hasta el 30 de septiembre de 2012, en el servicio de hospitalización de medicina interna del HUS, donde el paciente y el médico tratante aceptaran participar en el estudio de la pleuritis con los métodos estándares por ellos utilizados.

Los criterios de inclusión fueron: Pacientes mayores de 18 años de edad hospitalizados en el servicio de medicina interna del HUS durante el periodo del estudio, con derrame pleural tipo exudado linfocitario (relación proteínas del líquido sobre proteínas séricas mayor a 0.5 o relación LDH del líquido sobre LDH sérico mayor de 0.6 o LDH del líquido mayor de los 2/3 del límite superior del valor normal sérico y con más del 50% de linfocitos ^18^ sin etiología identificada, cuyo estudio haya incluido ZN de líquido o tejido pleural*,* cultivo de líquido o tejido pleural para *M. tuberculosis* y/o histología de tejido pleural con granulomas y en quienes se haya realizado RT-PCR con sondas de hibridación en líquido o tejido pleural buscando *M. tuberculosis.*

Los criterios de exclusión fueron: Derrame pleural tipo transudado (no cumple ninguno de los criterios de exudado expuestos anteriormente ^18^; Derrame pleural tipo exudado neutrofílico (cumple los criterios de exudado expuestos anteriormente y con más del 50% de neutrófilos); Pacientes a quienes las muestras se hayan procesado con técnicas diferentes a los protocolos que se describen más adelante en este documento. Pacientes en quienes el estudio de la etiología del derrame pleural haya quedado incompleto porque tuvieron resultados negativos en un número insuficiente de pruebas diagnósticas estándares para un exudado pleural linfocitario (no fueron estudiados con todas las pruebas que hacen parte del método de referencia de este trabajo a pesar de haber tenido resultados negativos en las pruebas que se les practicaron: ZN de líquido o tejido pleural*,* cultivo de líquido o tejido pleural para *M. tuberculosis* e histología de tejido pleural)

Inicialmente se utilizó la base de datos del laboratorio de biología molecular donde contamos con todas las RT-PCR sondas de hibridación para *M. tuberculosis* realizadas en líquido o tejido pleural durante los años del estudio y con esta información nos dispusimos a buscar (previa autorización del paciente y el hospital) en las historias clínicas de estos pacientes los demás criterios de inclusión requeridos.

Una vez recolectada la información, nos dispusimos a realizar su respectiva codificación y tabulación por doble entrada, para lo que se utilizó un instrumento diseñado para la recolección de datos.

### Métodos diagnósticos y método de referencia

Se definió un método de referencia combinado de la siguiente forma: 1. Cultivo de líquido o tejido pleural para *M. tuberculosis*; 2. Ziehl-Neelsen (ZN) de líquido o tejido pleural; e 3. Histología de tejido pleural. 

Con una de estas tres pruebas diagnósticas positiva se diagnosticó la pleuritis por *M. tuberculosis* y dicha entidad se descartó si las tres fueron negativas. Se consideró como caso probable si el método de referencia era negativo pero la RT-PCR sondas de hibridación para *M. tuberculosis* positiva y el ADA mayor de 47 UI/L ^6^. 

Tanto las pruebas del método de referencia como el ADA y la RT-PCR sondas de hibridación para *M. tuberculosis* fueron realizadas en paralelo una vez se hizo el diagnóstico a partir del exudado linfocitario inicialmente en líquido pleural y si estas no eran concluyentes para generar un diagnóstico definitivo se realizaron dichas pruebas de forma paralela en muestra de biopsia pleural. Al inicio del estudio no se tenía el diagnóstico de la etiología de dicho derrame pleural ni tampoco antecedente previo de TB ni cáncer, aunque esto no era un criterio de exclusión. 

La extracción total del ADN presente en el líquido pleural de los pacientes, así como el montaje de la RT-PCR sondas de hibridación se realizó según las recomendaciones del fabricante del kit (SACACE Biotechnologies, Italy). Para ello y con el fin de remover inhibidores de la reacción de la RT-PCR, inicialmente todos los líquidos pleurales obtenidos, fueron tratados con 250 mg de N-acetil-L Cisteina (Sigma), NaOH al 4% y citrato de sodio al 2.94%. El ADN posteriormente obtenido fue utilizado en el montaje de la RT-PCR, utilizando sondas de hibridación (Emisión FAM) para la detección cualitativa del gen IS6110 presente de forma específica en el ADN de las Micobacterias pertenecientes al complejo tuberculoso (*M. tuberculosis, M. africanum, M. bovis, M. bovis BCG, M. microti).* Bajo las siguientes condiciones de amplificación: 1 Ciclo: 95° C por 15 min, 40 Ciclos: 95° C por 15 s, 65° C por 30 s, 72° C por 15 s. Con el fin de descartar los falsos negativos por presencia de inhibidores de la reacción de amplificación, a todas las muestras se les adicionó un control interno (CI) de amplificación (Emisión JOE). El montaje de la RT-PCR y el análisis de los resultados se llevó acabo utilizando el equipo STRATAGENE MX 3005P en el programa MxPro. Las muestras fueron consideradas positivas o negativas basados en el aumento de la fluorescencia por encima del umbral establecido en los canales FAM y JOE. De esta forma, una muestra se consideró positiva para *M. tuberculosis* si la fluorescencia superó el límite del umbral establecido en el canal FAM (verde), mientras que una muestra se consideró negativa si no presentó fluorescencia en el canal FAM y presentó fluorescencia positiva en el canal JOE. Es importante aclarar que los resultados obtenidos para cada paciente con esta técnica se obtuvieron en un tiempo máximo de cuatro horas.

Todas las muestras incluidas en el estudio fueron cultivadas en medio sólido Ogawa kudot, utilizando la técnica de Petroff modificada, en donde las muestras liquidas fueron centrifugadas a 3,000 rpm durante 30 min y el sedimento es resuspendido en un máximo de 2 mL y depositado en el medio directamente; cultivando en posición inclinada a 37° C durante 8 semanas.

Estas pruebas fueron procesadas por personal del HUS con una amplia trayectoria y experiencia en biología molecular, microbiología, laboratorio clínico y patología, respectivamente. Las personas encargadas de ejecutar las pruebas del método de referencia desconocían el resultado de la RT-PCR para *M. tuberculosis*; y la persona encargada de realizar la RT-PCR para *M. tuberculosis* desconocía el resultado del método de referencia.

### Métodos estadísticos 

La estimación del tamaño muestral se hizo con base en las razones de verosimilitud e intervalos de confianza en un estudio de pruebas diagnósticas, de una sola muestra, donde las pruebas diagnósticas en evaluación son comparadas en los mismos sujetos (diseño emparejado), para una sensibilidad esperada del 90%, una especificidad esperada del 95% una prevalencia de tuberculosis pleural en la población estudiada del 50%, esperando una amplitud del intervalo de confianza del 95% de 0.1 a cada lado, utilizando la ecuación propuesta por Duffau ^19^^-^^21^. Con estos parámetros se estimó un tamaño de muestra para este estudio de 34 pacientes.

Se calcularon la sensibilidad, especificidad, valores predictivos positivo y negativo, y los respectivos intervalos de confianza, para la RT-PCR, cultivo y coloración de ZN en líquido pleural, comparados con el método de referencia. Los intervalos de confianza se determinaron mediante la Prueba Mid-P Exact. Se utilizó el software OpenEpi versión 2.3 (2009).

## Resultados

### Participantes y resultados 

Se obtuvo una población de 60 pacientes con derrame pleural en el HUS durante el periodo del estudio, de los cuales 40 cumplieron con los criterios de inclusión y de estos a nueve (prevalencia de 22.5%) se les confirmó el diagnóstico de TBP con el método de referencia. De estos nueve pacientes confirmados con TBP, 6 fueron positivos y 3 negativos para la RT-PCR, estos últimos, se consideraron como falsos negativos de la RT-PCR, ya que presentaron resolución del cuadro con el manejo antituberculoso. No hubo pacientes con TBP probable (TBP probable: método de referencia negativo con RT-PCR positiva y ADA mayor de 47UI/L). Se presentaron dos falsos positivos para la RT - PCR, uno con un mesotelioma pleural y el otro con una pleuritis crónica con hiperplasia mesotelial. En los pacientes con diagnóstico de TBP encontramos una media de 47.4 años de edad con una desviación estándar de (15.2. El 66.7% de los pacientes con TBP fueron hombres.

De los 40 pacientes incluidos en el estudio (Fig. 1), a 9 pacientes se les confirmó TBP: uno por cultivo para TB, ZN y PCR para TB positivos en liquido pleural; uno por ZN y PCR para TB positivos en liquido pleural; dos por cultivo para TB y PCR para TB positivos en liquido pleural; uno por cultivo para TB y PCR para TB positivos en liquido pleural y biopsia pleural con granulomas de caseificación; uno por cultivo para TB positivo en liquido pleural, cultivo para TB y PCR para TB positivos en tejido pleural y biopsia pleural con granulomas de caseificación; dos por biopsia pleural con granulomas de caseificación; uno por PCR para TB positiva en liquido pleural y biopsia pleural con granulomas de caseificación. A 31 pacientes se les descartó TBP por presentar el método de referencia negativo, encontrando como causa del derrame pleural linfocitario en el 29.0% asociación a neoplasia por biopsia pleural, de los cuales, tres casos fueron por mesotelioma y los restantes por metástasis, CA escamocelular y adenoma sin primario especificado. El 58.1% se asoció a inflamación crónica pleural reactiva sin otra etiología encontrada y el 12.9% no tuvo un diagnóstico claro al momento de la inclusión en el estudio. 

**Figura 1:** Diagrama de flujo: RT-PCR en líquido pleural para diagnóstico de TBP.

### Estimaciones

Para la prueba en estudio (RT-PCR cualitativa para *MTb*) se calculó una sensibilidad del 66.7% (IC 95%: 33.2%-90.7%) y una especificidad del 93.5% (IC 95%: 80.3%-98.9%). El VPP fue 75% (IC 95%: 38.8%-95.6%) y el VPN fue 90.6% (IC 95%: 76.6%-97.6%) (Tabla 1).

**Tabla 1:** Resultados en las diferentes pruebas diagnósticas con respecto al diagnóstico de TBP según el diagnóstico definitivo obtenido por el método de referencia.

Diagnóstico Definitivo	N: 40	RT - PCR en líquido		ZN líquido		ZN tejido		Cultivo MTb líquido		Cultivo MTb tejido		Histología pleura de TBP	
		Positiva	Negativa	Positiva	Negativa	Positiva	Negativa	Positiva	Negativa	Positiva	Negativa	Positiva	Negativa
TBP	9	6	3	2	3	-	2	5	2	1	-	5	-
Mesotelioma	5	1	4	-	5	-	-	-	5	-	-	-	5
Pleuritis crónica con hiperplasia mesotelial	2	1	1	-	2	-	1	-	2	-	-	-	2
Otros Tumores	5	-	5	-	5	-	-	-	5	-	-	-	5
Pleuritis inespecífica	19	-	19	-	17	-	2	-	18	-	1	-	19
Total	40	8	32	2	32	-	5	5	32	1	1	3	31

TBP: tuberculosis pleural; RT-PCR: reacción en cadena de la polimerasa en tiempo real; ZN: coloración de Ziehl Neelsen; MTb: Mycobacterium tuberculosis.

Con respecto al cultivo para *M. tuberculosis* en líquido pleural se encontró una sensibilidad del 55.6% (IC 95%: 39.7%-89.2%) con una especificidad del 100% (IC 95%: 87.1-100%); y para el ZN en líquido pleural se calculó una sensibilidad del 22.2% (IC 95%: 39.7%-89.2%) con una especificidad del 100% (IC 95%: 87.1% - 100%) (Tabla 1).

## Discusión

Con estos resultados se establecieron las características operativas de la RT -PCR para el diagnóstico de *M. tuberculosis* en líquido pleural en el HUS. Se determinó una sensibilidad (66.7%) similar a lo encontrado en estudios previamente referenciados ^12^^-^^14^^,^^16^. Por otra parte, la especificidad calculada fue relativamente alta (93.5%) y ambos resultados fueron similares a lo encontrado por otros autores. 

En el cultivo para *M. tuberculosis* en líquido pleural se encontró una sensibilidad del 55.6% mientras que para el ZN en líquido pleural fue del 22.2%, ambos tuvieron una especificidad del 100%. Estos valores muestran sensibilidades menores a la de la RT-PCR, confirmando una vez más la necesidad de tener un método de referencia combinado ya que estas pruebas por si solas no podrían descartar la presencia de tuberculosis pleural. Adicionalmente, en el caso de los cultivos, el tiempo necesario para obtener el diagnóstico se alarga hasta 8 semanas de tal manera que obliga a recurrir a una prueba rápida, como la RT-PCR, para definir el manejo inicial del paciente en un tiempo máximo de cuatro horas que es lo que esta prueba se tardaría en generar un resultado.

En el meta-análisis realizado por Pai*, et al.* ^12^*,* se evaluó PCR comerciales y artesanales, reportando una sensibilidad del 62% y 71%, con una especificidad del 98% y 93%, respectivamente, lo cual es similar a lo encontrado en nuestra investigación para RT-PCR. Los estudios incluidos en este meta-análisis tenían tamaños muestrales pequeños y eran heterogéneos, los método de referencia utilizados incluían cultivo para *M. tuberculosis*, hallazgos clínicos, microbiología o biopsia, de tal forma que estos método de referencia carecen de precisión para dar un diagnóstico definitivo y dificultan la validación de la prueba en estudio. Posteriormente en el año 2005, Chakravorty *et al*. ^13^, utilizaron el ensayo IS6110 y devRf3 (pruebas artesanales) en líquido con una sensibilidad del 75.5% y una especificidad del 93.8%, similar para cada prueba, con una mayor sensibilidad a la reportada por nosotros e igual especificidad, con un método de referencia compuesto, no bien definido, dado por la clínica, microbiología, cito/histología y la respuesta a tratamiento, con una muestra de 87 pacientes. En Taiwán, Liu *et al.* ^14^, publicaron un estudio de PCR artesanal utilizando el segmento IS6110, reportando una sensibilidad del 43.3% y una especificidad del 95.5%, siendo la sensibilidad inferior a la descrita en nuestra investigación, sin variaciones en la especificidad y con falsos positivos en un 4.5% de los pacientes estudiados. Adicionalmente en el método de referencia combinado se incluyó la clínica y la respuesta al tratamiento empírico lo que dificulta realizar una evaluación objetiva. Y finalmente Kalantri, *et al*. ^16^, con una población de 204 casos divididos en tres grupos por su forma de diagnóstico (confirmada, probable y sin TBP) reportó una sensibilidad del 80% al tener un método de referencia compuesto igual al usado por nosotros y del 57.7% al tener en cuenta la clínica, respuesta al tratamiento y haber excluido otras patologías, con una especificidad del 98.0% similar a la descrita en el presente trabajo. 

De acuerdo con nuestro método de referencia, en nuestro estudio tuvimos 9 casos de TBP y 31 casos con pleuritis linfocitaria por causas diferentes a TB, lo cual nos da una prevalencia de TBP de 23%, esta prevalencia es similar a la encontrada en estudios de TBP en España ^12^ y superior a la prevalencia de TBP en los estudios realizados en la mayoría de países con PIB superior al de nuestro país ^12^ y podría explicarse por una transición epidemiológica en nuestra población con un incremento en la frecuencia de enfermedades neoplásicas y una reducción en la frecuencia de enfermedades infecciosas.

Las características operativas de la PCR varían dependiendo de la técnica utilizada y de la asociación de otras pruebas diagnósticas y características clínicas del paciente. Se puede observar que el uso de la RT-PCR requiere menor tiempo, con alta especificidad pero con baja sensibilidad. Esto hace que la prueba resulte insuficiente para descartar TBP si la RT-PCR es negativa y no existe otro diagnóstico alterno, y exige realizar el método de referencia combinado completo. Al tener unos valores de sensibilidad y especificidad superiores a los obtenidos con las otras pruebas en forma individual y arrojar resultados en forma mucho más rápida, resulta una prueba útil para definir tempranamente el manejo a seguir con un paciente que tenga exudado linfocitario: si es positiva habría razones válidas para comenzar el tratamiento anti -tuberculoso y si es negativa para no hacerlo. Sin embargo estos resultados deben ser analizados en el contexto clínico del paciente y deben confirmarse con el método de referencia (ZN, cultivo y biopsia), ya que los valores predictivos no son suficientemente buenos (VPP: 75.0% y VPN: 90.6%) para usar la RT-PCR como un método diagnóstico definitivo; y, de hecho, se presentaron dos falsos positivos en donde el diagnóstico fue mesotelioma y pleuritis crónica con hiperplasia mesotelial, respectivamente.

Para el presente estudio se utilizó un método de referencia combinado lo que permitió tener un diagnóstico definitivo y objetivo de TBP, y de esta manera hacer una evaluación más precisa de las características operativas de la prueba en estudio, convirtiéndose en una fortaleza para la presente investigación. Sin embargo, debemos señalar que como debilidad del estudio está el tamaño muestral, que resultó bajo para los hallazgos finalmente encontrados, lo cual aumentó los intervalos de confianza y disminuyó la precisión del estudio. Esto nos obliga a sugerir la realización de estudios de validación de la RT-PCR con mayores tamaños muestrales, para aumentar la precisión de los resultados.

## Conclusiones

La RT-PCR para *M. tuberculosis* en líquido pleural tiene una buena especificidad para el diagnóstico de TBP, pero debido a que sus valores predictivos no son suficientemente altos, no debe utilizarse para el diagnóstico definitivo de la enfermedad. Su mayor utilidad sería como método para definir rápidamente la conducta a seguir con un paciente, mientras llegan los reportes de las pruebas diagnósticas definitivas. Los cultivos para *M. tuberculosis* y los ZN de líquido y tejido pleural tienen menor rendimiento diagnóstico que la RT-PCR y es necesario seguir usando el método de referencia combinado para establecer el diagnóstico definitivo de la pleuritis linfocitaria. 

Es necesario realizar nuevos estudios con un mayor tamaño muestral con el fin de aumentar la precisión de los resultados previamente establecidos.
